# Serious psychological distress among slum dwellers and unhoused people in Ho Chi Minh City, Vietnam: a pilot study

**DOI:** 10.1186/s41182-025-00729-1

**Published:** 2025-04-14

**Authors:** Hitoshi Murakami, Nguyen Thuy Linh, Masami Fujita, Lam Ngoc Thuy, Nguyen Hong Phuc, Kieu Thi Mai Huong, Le Tuan Anh, Pham Thi Ngoc Mai, Khuat Thi Hai Oanh

**Affiliations:** 1 Bureau of International Health Cooperation, National Center for Global Health and Medicine, 1-21-1 Toyama, Shinjuku-ku, Tokyo, 162-8655 Japan; 2 Center for Supporting Community Development Initiative (SCDI), No.9, Alley 165/30 Thai Ha Street, Lang Ha Ward, Dong Da District, Hanoi, Vietnam; 3 School of Tropical Medicine and Global Health, Nagasaki University, 1-12-4 Sakamoto, Nagasaki, 852-8523 Japan

**Keywords:** COVID-19, Unstable housing, Urban health, Urban slum, Slum dwellers, Unhoused, Homelessness, Serious psychological distress

## Abstract

**Background:**

Mental health is reported to be a significant issue among slum dwellers and unhoused (homeless) individuals worldwide, particularly those facing housing instability. Ho Chi Minh City, the largest city and industrial hub of Vietnam, has a substantial population experiencing housing instability, although its exact scale has rarely been accurately measured. This study aimed to estimate the prevalence of serious psychological distress among slum dwellers and unhoused individuals in Ho Chi Minh City and to identify factors associated with serious psychological distress.

**Methods:**

A cross-sectional survey involving 415 individuals experiencing housing instability, including 383 slum dwellers and 32 unhoused individuals, was conducted between November 2023 and April 2024. Data were collected using a structured questionnaire incorporating the 6-item version of the Kessler Psychological Distress Scale (K6).

**Results:**

The overall prevalence of serious psychological distress was 19.8%, with 18.5% among slum dwellers and 34.4% among unhoused people. Multiple logistic regression analysis revealed significant associations between serious psychological distress and female gender (adjusted odds ratio = 3.086, *p* < 0.001), labour exploitation (adjusted odds ratio = 1.914, *p* = 0.046), and debt (adjusted odds ratio = 3.109, *p* < 0.001). Notably, 68.7% of the participants reported experiencing some form of labour exploitation, which commonly included contract rejections, forced overwork, wage theft, and physical or verbal abuse. Furthermore, 43.7% of the participants were in debt, with 38.6% borrowing from moneylenders.

**Conclusions:**

The prevalence of serious psychological distress among individuals with unstable housing in Ho Chi Minh City was significantly higher than that of the general population, highlighting the urgent need for mental health interventions targeted at this population. Those with serious psychological distress frequently faced both labour and economic exploitation, without sufficient social protection. In terms of labour exploitation, policy interventions, particularly from an occupational health perspective, are necessary. To address economic exploitation through debt, given the prevalence of loan sharks, efforts to crack down on predatory lending and promote financial inclusion are essential.

## Background

Housing is one of the major social determinants of health [1–4]. Unstable housing, whether among migrants or domestic residents, is known to be associated with mental health issues, including psychological distress, depression, anxiety, and suicide [5–10]. Poverty manifests itself on multiple levels, ranging from income inequality at the macro level to precarious housing, food insecurity, and malnutrition at the micro level [11, 12]. In fact, housing insecurity is not an isolated phenomenon but is interlinked with other deprivations stemming from poverty, such as food insecurity, energy poverty, and unemployment [13].

Mental health is reported to be a significant issue among slum dwellers and unhoused (homeless) individuals worldwide, particularly those facing housing instability. Systematic reviews have identified mental health as a significant issue among slum dwellers [14, 15]. Various social determinants of health related to slum living, such as socioeconomic status, gender, living conditions, food insecurity, social capital, social support [14], healthcare access and utilisation [15], housing, nutrition, neighbourhood characteristics, occupational factors, and health behaviours [16] have been highlighted. The prevalence of mental disorders among unhoused populations has been derived from systematic reviews and meta-analyses [17]. The pooled prevalence of mental disorders among unhoused individuals was estimated to be 9% in the United States, 12% in Western Europe, 19% in the United Kingdom, and 16% in Australia [18]. A systematic review of psychopathology among unhoused youth revealed that the prevalence of disorders listed in the Diagnostic and Statistical Manual of Mental Disorders (DSM) and International Classification of Diseases (ICD) ranged from 48.4 to 98% [19].

Psychological distress (PD) is generally defined as a state of emotional suffering characterised by symptoms of depression (e.g., loss of interest, sadness, hopelessness) and anxiety (e.g., restlessness, tension) [20]. Although PD is often described as a nonspecific mental health issue [20, 21], it is clearly characterised by symptoms of depression and anxiety. Thus, while PD and these mental disorders are distinct phenomena, they are not entirely separate [22]. Scales measuring PD, such as the Kessler Psychological Distress Scales (K6/K10), have been reported to most effectively detect major depression and dysthymia according to the fourth edition of the DSM (DSM-IV) [23, 24]. Serious psychological distress (SPD) includes mental health problems severe enough to cause moderate-to-serious impairment in social, occupational, or school functioning and to require treatment [25, 26].

Ho Chi Minh City (HCMC) is the largest city in Vietnam, with a population of 9,456,700 as of 2023 [27], and serves as the country’s industrial centre. Unstable housing has been recognised as a significant issue in HCMC for decades, but its exact scale has seldom been accurately measured [28]. In 2004, approximately 15% of the city's housing consisted of slums or squatter settlements. In 2002, municipal authorities registered 67,000 households living in slums [29]. These informal areas are primarily inhabited by low-income households, many of whom live in precarious conditions without adequate sanitation or housing stability. The city's rapid urbanisation, with thousands of new migrants arriving each year, has strained housing resources and contributed to the expansion of these settlements [30]. In 1995, it was estimated that 5% of the city's population was unhoused [31, 32].

There is a clear need for a study addressing mental health issues among slum dwellers and unhoused individuals in HCMC, which is facing a pressing issue of housing instability, as mentioned in the previous paragraph. While mental health concerns among slum dwellers and unhoused individuals have been highlighted globally, to the best of the authors’ knowledge, no research on the mental health status of slum dwellers or unhoused individuals in Vietnam, including HCMC, has been published to date. This clearly highlights a significant research gap that needs to be addressed. Establishing the magnitude of mental health problems and identifying the social determinants of health associated with them are essential for formulating effective public health policies, intervention programs, and healthcare plans for these highly marginalised and extremely hard-to-reach urban populations.

Building on the above background, the present study aimed to: (1) examine socioeconomic vulnerabilities, (2) estimate the prevalence of SPD, and (3) identify social determinants of health significantly associated with SPD among populations with unstable housing, including slum dwellers and unhoused individuals, in HCMC, Vietnam. The study was conducted by the Center for Supporting Community Development Initiatives (SCDI), Vietnam, in collaboration with the National Center for Global Health and Medicine (NCGM), Japan. SCDI is a nonprofit organisation established in 2010, with the mission of improving the quality of life and promoting social inclusion for vulnerable and marginalised populations in Vietnam. SCDI has been actively supporting those with unstable housing, including slum dwellers and the unhoused population, in HCMC.

## Methods

### Study design and participants

This study is a cross-sectional survey. The target population of the study comprised individuals with unstable housing, including those living in slums or unhoused, who were accessed by the SCDI’s outreach team or who sought drop-in services in HCMC. The inclusion criteria were as follows: (1) individuals present during visits for listing for sampling, (2) those aged 18 years or older, and (3) those who agreed to participate in the survey. The exclusion criteria was individuals who could not understand the explanation document or communicate verbally and clearly with the research team. The slum-dwelling participants were recruited from 13 slum sites serviced by the SCDI, located across ten wards in four of the 16 urban districts of HCMC. These sites included specific alleys, areas around hostels, and settlements of garbage scavengers. Unhoused people were defined as those who had slept in places other than their homes over the past 30 days. This included individuals sleeping in public spaces, such as sidewalks, bus stations, markets, under bridges, and parks, as well as in workplaces, hammock cafés, and internet cafés.

### Sampling

Slum dwellers residing in 13 slum sites serviced by the SCDI were pre-visited by local collaborators. A comprehensive sampling frame was not established prior to these pre-visits, as only 77% of residents were registered at their living locations. Instead, pre-visits were conducted based on local collaborators’ knowledge of individuals residing in the target slums who were 18 years or older, with the aim of covering all eligible participants. Those who expressed willingness to participate were listed. A total of 460 slum dwellers were listed, and all were subsequently visited by the survey teams. As a result, 383 individuals actually participated, yielding a response rate of 83.3% at this stage. The primary reason for non-participation was their absence from their residences at the time of the survey, often due to visits outside HCMC, reflecting their highly mobile lifestyle. According to local demographic statistics, the entire population of individuals aged 18 or older residing in non-permanent housing in the ten wards hosting these 13 sites was estimated at 1,792. Thus, those listed represent approximately 26% of this population.

For unhoused individuals, all those contacted by the SCDI team—either through outreach or drop-in services—who agreed to participate in the study were included; thus, a non-random sampling approach was used. In total, 415 individuals with unstable housing, comprising 383 slum dwellers and 32 unhoused individuals, were selected for the study. All participants were once again provided with a written explanatory sheet and asked to confirm their willingness to participate by providing written informed consent at the time of the actual survey. Notably, none of them refused participation.

### Data collection

The data were collected through face-to-face interviews. A structured questionnaire was used, which included the 6-item version of the Kessler Psychological Distress Scale (K6) to identify SPD and questions to elaborate on the following statuses: basic sociodemographic information (age, gender, school years, whether the individual is a migrant or was born in HCMC, marital status, household size, and the number of children by age group), basic socioeconomic status (means of earning a living, monthly income, ownership of a private house, and sleeping location in the last 30 days), entitlement to social support due to designated vulnerability status, cash or in-kind support received, identification and registration, health insurance coverage, experiences of labour exploitation, incarceration, detention, debt, the impact of and social protection during the COVID-19 pandemic, and human immunodeficiency virus (HIV) and tuberculosis status. The questionnaire was pretested on several slum dwellers and unhoused people, and unclear expressions were revised for clarity before being finalised. The K6 questions were kept in their original Vietnamese version. The survey was conducted with the recruitment period starting on 15 November 2023 and ending on 6 April 2024. The SCDI staff and other trained interviewers administered the questionnaire independently of the local collaborators.

### Measurement of serious psychological distress (SPD)

SPD was assessed using the K6 scale, a brief, 6-item self-report tool that evaluates general psychological distress experienced over the past 30 days. The scale includes questions about feelings of nervousness, hopelessness, restlessness, depression that cannot be alleviated, excessive effort in daily activities, and feelings of worthlessness. Each item is scored on a 5-point Likert scale, ranging from 0 (none of the time) to 4 (all of the time), yielding a total score between 0 and 24. Higher scores indicate greater distress. SPD was defined as a K6 score of ≥ 13 [33, 34]. The K6 is widely recognised for its brevity, reliability, and validity in distinguishing cases that are consistent with DSM-IV diagnoses [35]. The Vietnamese version of the K6 has been validated, demonstrating reliable internal consistency and strong construct validity [36]. In our survey data, the Cronbach’s alpha of the K6 was 0.863, indicating a high level of internal consistency.

### Statistical analysis

We estimated the prevalence of SPD and calculated the proportion of individuals with various socioeconomic vulnerabilities, including possession of an identification (ID) card, registration at their place of residence, possession of a health insurance card, experiences of any form of labour exploitation, and debt status, using univariate analyses. We then explored the associations between SPD and various explanatory variables presented in the “Data Collection” section. Chi-square tests were conducted for categorical variables, and *t* tests were applied for continuous variables (age, household size, number of children, and monthly income). We constructed a multiple logistic regression model, with SPD as the dependent variable and the explanatory variables that showed significant associations with SPD as independent variables. The dependent variable, SPD, was dichotomous, with categories of SPD (coded as 1) and non-SPD (coded as 0). Statistical significance was set at *p* < 0.05. We applied a combination of bivariate analysis and multivariate logistic regression analysis for two reasons. First, bivariate analysis helps screen which explanatory variables may be associated with the dependent (outcome) variable. Second, it improves the reliability of the analysis, because if an association observed in bivariate analysis remains significant in multivariate analysis, it strengthens the validity of the findings.

Since the explanatory variables excluded during the bivariate analyses may become important predictors of SPD when combined with other variables, we reintroduced them one by one into the preliminary model to assess their *p* values and their potential confounding effects by examining changes in the regression coefficients (*β*) of the key exposure variables, namely, labour exploitation and indebtedness. We set a 15% change in β as the cutoff value to determine whether the reintroduced variables acted as confounders [37, 38]. In addition, we monitored changes in model performance using the area under the ROC curve (AUC), which measures the model’s ability to discriminate the outcome, and Nagelkerke’s *R*^2^, which represents model fit. As a result, none of the previously excluded independent variables showed statistical significance (*p* < 0.05). Only one variables—“living with an intimate partner”—met the criteria for confounding, but we decided not to include as it remained non-significant. No variable significantly altered the AUC. Only “housemaid” as a job and “ever been incarcerated” increased Nagelkerke’s *R*^2^ by more than 5%, but they remained non-significant after reintegration.

Data analyses were performed using the Statistical Package for the Social Sciences (SPSS) version 27 (IBM Corp., Armonk, NY, USA).

### Ethical considerations

The study protocol, explanation document with the informed consent form, consent withdrawal form, and questionnaire were reviewed and approved by both the NCGM Ethics Independent Review Board (IRB) on 9 July 2023 (NCGM-S-004698-00), and the Ethics IRB of the Institute for Social Development Studies (ISDS), Vietnam, on 28 September 2023, before data collection commenced. Written informed consent with participants’ signatures was obtained from all participants prior to their involvement in the study. The explanation document included the purpose, methods, risks, and benefits of the study. The participants were informed that consent was at their discretion, that they would not be treated unfavourably if they chose not to participate, and that they could withdraw their consent at any time, even after the data collection. Copies of the explanation document and consent forms were provided to the participants, while the original consent forms were retained by the SCDI. All researchers involved in this study adhered to the Declaration of Helsinki (Revised 2013 Fortaleza) and conducted the research in accordance with the "Ethical Guidelines for Medical and Biological Research Involving Human Participants" set forth by the Ministry of Health, Labour and Welfare (MOHLW), the Ministry of Education, Culture, Sports, Science and Technology (MEXT), and the Ministry of Economy, Trade and Industry (METI) of Japan.

## Results

### Basic characteristics of the participants

Table 1 presents the basic social, demographic, and economic characteristics of the 415 study participants. The average age was 44.12 years (standard deviation or SD: 0.65). The gender distribution was female-dominant, with females accounting for 244 participants (58.8%). A total of 383 participants (92.3%) were slum dwellers, while 32 participants (7.7%) were unhoused. Among the participants, 219 (52.8%) were migrants from other provinces, whereas 196 (47.2%) were born in HCMC. The average duration of schooling was 5.93 years. In addition, 101 participants (24.3%) were married, and 79 (19.0%) were cohabiting without formal marriage. The average household size was 4.06 persons (SD: 1.90). The average monthly income was equivalent to 134.45 US dollars (SD: 122.30) based on the exchange rate as of 1 February 2024, the midpoint of the survey. Regarding the means of earning a living, 104 participants (25.1%) had no income and were dependent on others; 85 (20.4%) were engaged in informal employment; 58 (14.0%) were garbage collectors; 39 (9.4%) were motorcycle drivers or shippers; 29 (7.0%) were lottery ticket sellers; and 26 (6.3%) were street vendors.

**Table 1:** Basic characteristics of the study participants

Characteristics	Figures
Total number of study participants	415
Age, mean (SD) in years	44.12 (0.65)
Female gender, *N* (%)	244 (58.8)
*Housing status*	
Slum dweller, *N* (%)	383 (92.3)
Unhoused, *N* (%)	32 (7.7)
*Migrant or born in HCMC*	
Migrant, *N* (%)	219 (52.8)
Born in HCMC, *N* (%)	196 (47.2)
Duration of schooling, mean (SD) in years	5.93 (3.86)
*Marital status*	
Married, *N* (%)	101 (24.3)
Cohabiting without formal marriage, *N* (%)	79 (19.0)
Separated, divorced or widowed, *N* (%)	37 (8.9)
Never married, *N* (%)	101 (24.3)
Household size, mean (SD) in persons	4.06 (1.90)
Monthly income, mean (SD) in US dollars*	134.45 (122.30)
*Means of earning a living*	
No income and dependent on others, *N* (%)	104 (25.1)
Informal employment, *N* (%)	85 (20.4)
Garbage collector, *N* (%)	58 (14.0)
Motorcycle driver/shipper, *N* (%)	39 (9.4)
Lottery ticket seller, *N* (%)	29 (7.0)
Street vender, *N* (%)	26 (6.3)
Housemaid, *N* (%)	13 (3.1)
Construction worker, *N* (%)	12 (2.9)
Shopkeeper, *N* (%)	12 (2.9)
Formal employment, *N* (%)	10 (2.4)
Others, *N* (%)	27 (6.5)

*SD* standard deviation, *N* number, *HCMC* Ho Chi Minh City

^*^Based on the exchange rate as of 1 February 2024

### Status of socioeconomic vulnerabilities

Table 2 presents the socioeconomic vulnerabilities of the study participants, with a breakdown between slum dwellers and unhoused people. A lack of civil registration, evident in the absence of ID cards and registration at their living locations, was more prevalent among the unhoused population (37.5% and 81.3%) than among the slum dwellers (9.7% and 23.0%). Health insurance coverage extended to only 57.8% of the participants and only 34.4% of the unhoused people. A total of 68.9% experienced some form of labour exploitation, and 43.6% were in debt.

**Table 2:** Status of socioeconomic vulnerabilities of the 415 study participants

	No. of participants	Prevalence (%)	95% CI
*Lack of ID card*			
All participants	49/415	11.8	8.7–14.9
Slum dwellers	37/383	9.7	6.7–12.6
Unhoused people	12/32	37.5	19.8–55.2
*Lack of registration at living location*			
All participants	114/415	27.5	23.2–31.8
Slum dwellers	88/383	23	18.7–27.2
Unhoused people	26/32	81.3	67.0–95.5
*Lack of health insurance coverage*			
All participants	175/415	42.2	37.4–46.9
Slum dwellers	154/383	40.2	35.3–45.1
Unhoused people	21/32	65.6	48.2–83.0
*Faced any form of labour exploitation*			
All participants	286/415	68.9	64.4–73.4
Slum dwellers	264/383	68.9	64.3–73.6
Unhoused people	22/32	68.8	51.8–85.7
*In debt*			
All participants	181/415	43.6	38.8–48.4
Slum dwellers	168/383	43.9	38.9–48.9
Unhoused people	13/32	40.6	22.6–58.6

*ID* identification, *CI* confidence interval

Among the 286 participants who experienced labour exploitation, 211 (73.8%) faced contract rejections, 83 (29.0%) were forced to work excessively hard, 75 (26.2%) experienced wage theft, 53 (18.5%) suffered physical or verbal abuse, and 29 (10.1%) encountered work conditions that did not align with what was promised. Among the 181 participants who were in debt, 160 (88.4%) borrowed from money lenders with interest, whereas 21 (11.6%) borrowed from acquaintances without interest. Regarding the reasons for being in debt, 94 (51.9%) borrowed for rent payments, 89 (49.2%) for purchasing food, 72 (39.8%) for covering electricity and water bills, 37 (20.4%) for medical treatments, and 34 (18.8%) for educational expenses for their children.

### Estimated prevalence of serious psychological distress (SPD)

As shown in Table 3, the estimated prevalence of SPD, defined as a K6 score of ≥ 13, among all 415 participants was 19.8%. The prevalence among slum dwellers was 18.5%, whereas among unhoused people, it was 34.4%. The estimated prevalence of moderate to severe PD, defined as a K6 score of ≥ 8, was 38.8% among all participants, 38.1% among slum dwellers, and 46.9% among unhoused people.

**Table 3:** Prevalence of serious psychological distress (SPD) among all participants, slum dwellers, and unhoused people

	No. of participants with SPD	Prevalence (%)	95% CI
All participants	82/415	19.8	16.0–23.9
Slum dwellers	71/383	18.5	14.8–22.8
Unhoused people	11/32	34.4	18.6–53.2

*CI* confidence interval

### Factors associated with serious psychological distress (SPD)

Table 4 summarizes the bivariate analyses of the associations between SPD and various social, demographic, and economic explanatory variables. The analyses revealed statistically significant associations between SPD and ten explanatory variables. Among the sociodemographic factors, female gender (odds ratio or OR = 2.572, *p* < 0.001), being unhoused (OR = 2.302, *p* = 0.031), and sleeping in public places (such as on pavements, at bus stations, in markets, or under bridges/parks) (OR = 5.892, *p* < 0.001) were significantly associated with SPD. Among the vulnerability factors, individuals living with HIV who can no longer work (OR = 5.641, *p* = 0.012), those meeting vulnerability criteria for monthly cash social assistance (OR = 2.711, *p* = 0.011), those who have experienced any form of labour exploitation (OR = 1.938, *p* = 0.024), and those currently in debt (OR = *p* < 0.001) were significantly associated with SPD. In terms of civil registration status and public service entitlements, a lack of registration at the current place of residence (OR = 1.959, *p* = 0.009), a lack of an ID card (OR = 2.740, *p* = 0.001), and a lack of a valid health insurance card (OR = 1.785, *p* = 0.019) were significantly associated with SPD. We examined multicollinearity among these ten independent variables by calculating Spearman’s correlation coefficients (r) for each pair. No combination, including “living with HIV and unable to work” and “meeting the criteria for monthly cash social assistance” or “being unhoused” and “sleeping in public places,” showed an *r* > 0.80.

**Table 4:** Bivariate analyses of associations between serious psychological distress (SPD) and various explanatory variables

Explanatory variables	*N*	Chi^2^	OR (95%CI)	*P*
*1. Sociodemographic factors*				
Female gender	244	11.93	2.572 (1.487–4.450)	0.001*
Being unhoused	32	4.67	2.302 (1.062–4.990)	0.031*
Sleeping in public places	14	12.77	5.892 (1.985–17.492)	0.000*
*2. Vulnerability factors*				
PLH who can no longer work	7	6.28	5.641 (1.237–25.719)	0.012*
Meeting vulnerability criteria for monthly cash social assistance	29	6.49	2.711 (1.227–5.992)	0.011*
Faced labour exploitation	286	5.11	1.938 (1.085–3.462)	0.024*
Being currently in debt	181	20.55	3.128 (1.884–5.195)	0.000*
*3. Civil registration status and public service entitlement*				
Lack of registration at living location	114	6.85	1.959 (1.178 3.259)	0.009*
Lack of ID card	49	10.10	2.740 (1.444–5.198)	0.001*
Lack of health insurance coverage	175	5.53	1.785 (1.098–2.904)	0.019*

*N* number, *PLH* people living with HIV (human immunodeficiency virus), *ID* identification, *OR* odds ratio, *CI* confidence interval

^*^P value < 0.05, with statistical significance also highlighted by bold letters

Table 5 shows the multiple logistic regression model, which designated SPD as the dependent (outcome) variable and included the ten variables that showed statistically significant associations with SPD in the bivariate analysis (*p* < 0.05) as independent (explanatory) variables. It revealed that only female gender (*p* < 0.001), experience of any form of labour exploitation (*p* = 0.046), and being currently in debt (*p* < 0.001) remained significantly associated with SPD (*p* < 0.05) after adjusting for confounding.

**Table 5:** Multivariate logistic regression analysis of associations between serious psychological distress (SPD) and various explanatory variables

Explanatory variables	*N*	*β*	AOR (95%CI)	*P*
*1. Sociodemographic factors*				
Female gender	244	1.127	3.086 (1.670–5.702)	0.000*
Being unhoused	32	0.338	1.403 (0.351–5.601)	0.632
Sleeping in public places	14	1.389	4.013 (0.745–21.613)	0.106
*2. Vulnerability factors*				
PLH who can no longer work	7	1.752	5.769 (0.730–45.610)	0.097
Meeting vulnerability criteria for monthly cash social assistance	29	0.695	2.003 (0.713–5.628)	0.187
Faced labour exploitation	286	0.649	1.914 (1.011–3.625)	0.046*
Being currently in debt	181	1.134	3.109 (1.787–5.409)	0.000*
*3. Civil registration status and public service entitlement*				
Lack of registration at living location	114	0.148	1.160 (0.624–2.157)	0.640
Lack of ID card	49	0.639	1.895 (0.855–4.201)	0.116
Lack of health insurance coverage	175	0.315	1.370 (0.766–2.449)	0.288

*N* number, *β* regression coefficients, *PLH* people living with HIV (human immunodeficiency virus), *ID* identification, *AOR* adjusted odds ratio, *CI* confidence interval

^*^P value < 0.05, with statistical significance also highlighted by bold letters

## Discussion

In response to the objectives of examining socioeconomic vulnerabilities, estimating the prevalence of SPD, and identifying factors associated with SPD among populations with unstable housing, including slum dwellers and unhoused individuals in HCMC, Vietnam, the present study revealed precarious civil registration statuses and limited public service entitlement among the participants. The study estimated the prevalence of SPD at 19.8% (18.5% among slum dwellers and 34.4% among unhoused people) and identified female gender, labour exploitation, and indebtedness as factors significantly associated with SPD. These findings make a substantial contribution to both the service delivery and advocacy policies of SCDI and local authorities.

The socioeconomic status of our participants, relative to both national and HCMC standards, was generally disadvantaged. The average monthly income of our participants was 134.45 US dollars, equivalent to 3,279,164 Vietnam dong (VND), which was approximately 66.1% of the national average of 4,962,000 VND and 50.3% of the HCMC average of 6,518,000 VND in 2023 [27]. The income poverty rate of our participants, which is based on the national urban poverty line, was 37.8%, with almost no difference between slum dwellers and unhoused people, whereas the nationwide income poverty rate was 4.3% in 2022 [39]. The informal employment rate among our participants, at 96.8%, far exceeded the national rate of 65.8% and HCMC’s 46.6% in 2023 [27]. In addition, the unemployment rate among our participants, at 25.1%, was significantly higher than both the national average of 2.34% and HCMC's 4.19% in 2023 [27].

The precarity of civil registration statuses and limited entitlement to public services among slum dwellers in HCMC has been highlighted by existing literature. A 2012 study of 500 slum households in HCMC described their difficulties obtaining household registration, land use certificates, and housing certificates due to their temporary residential status. This situation has led to low and unstable incomes, as well as predominantly informal, low-skilled, and precarious employment [28]. The majority of slum dwellers lack legal residential papers, affecting both land tenure and access to public services [40].

The socioeconomic vulnerabilities were even more pronounced among the unhoused people, a finding supported by existing literature. One contributing factor to their unstable lives is the relocation policy. In 2020, the authorities of HCMC began gathering and sending unhoused individuals to the HCMC Social Support Center as a measure against the COVID-19 pandemic, with 1500–2000 unhoused people sent to the Center annually [41, 42]. Unhoused individuals hiding from authorities were excluded from the list of beneficiaries of the government’s second support package in response to COVID-19 [43]. Several studies have shown that discrimination is associated with PD [20, 44, 45].

The SPD prevalence of 19.8% among our participants (18.5% among slum dwellers and 34.4% among unhoused people) was significantly higher than that reported in the general population of various countries. In the United States (US), analyses of National Health Interview Survey (NHIS) data from different periods between 1997 and 2018 revealed that the SPD prevalence among the noninstitutionalised adult population, as measured by the K6 scale, ranged from 2.7 to 3.4% [25, 26, 46, 47]. A comprehensive survey on living conditions in Japan from 2007 to 2016 revealed that the SPD prevalence among adults, measured by the K6, ranged from 4.0 to 4.2% [48]. Various online surveys conducted in Japan during the early phase of the COVID-19 pandemic in 2020 showed SPD prevalence in the general population, as measured by K6, ranged from 9.2 to 11.2% [49–52]. The Household, Income and Labor Dynamics in Australia (HILDA) Survey indicated that the SPD prevalence among adults, measured by K10—the 10-item version of the Kessler Psychological Distress Scale—increased from 4.8% in 2007 to 7.4% in 2017 [53]. The higher prevalence of SPD among our participants than among the general public of various countries can be interpreted as a reflection of the chronic stress caused by housing precarity.

Published studies on the prevalence of SPD among the Vietnamese population are very limited. However, the prevalence of SPD among Vietnamese students in the US, as measured by the K10 scale, was 18.5%, which is comparable to the rate observed in our sample of slum dwellers [54]. The prevalence of PD, not limited to SPD, in the general Vietnamese population of a mountainous province, as measured by the K6 scale, was reported to be 38.2% [55]. The estimated prevalence of moderate to severe PD, defined as a K6 score of ≥ 8, was 38.8% among our participants, which is nearly identical to the reported figure. However, the prevalence among unhoused individuals was significantly higher at 46.9%. The comparable levels of SPD prevalence among our participants and those in the previous studies likely indicate a similar degree of stress among Vietnamese people with precarious housing, studying abroad, and residing in mountainous regions.

High PD/SPD prevalence among slum dwellers has been reported worldwide. The SPD prevalence among slum dwellers in Port-au-Prince, Haiti, as measured by the K6, was 24.1%, which was even higher than that in our slum dweller sample, while the PD prevalence was 86.5%. Female gender and increasing age were associated with SPD [56]. In São Paulo, Brazil, the PD prevalence among slum dwellers, as measured by the General Health Questionnaire-12 (GHQ-12), was 85.0%, with food insecurity and low income being key contributors to PD [57]. The higher SPD/PD prevalence among slum dwellers in Haiti and Brazil compared to our participants likely reflects the differing living conditions across various mega-cities.

Elevated levels of PD/SPD among unhoused people are also highlighted in the existing literature. The SPD prevalence among mothers who were unhoused and entered shelters between 2010 and 2012 in major US cities, as measured by the K6, was 22.0% [58]. In northern California, PD prevalence among unhoused adults, as measured by the Center for Epidemiological Studies Depression Scale (CES-D), decreased from 63.7% to 49.4% over a follow-up period of 3 months to 1 year. Securing their own apartment during the follow-up period significantly reduced PD prevalence [59]. In the Netherlands, the prevalence of high levels of PD among unhoused people in four major cities, as measured by the Brief Symptom Inventory-18 (BSI-18), was 39.5%, which decreased to 27.0% after entering a social relief system. Meeting care needs, health insurance coverage, social support from family, and a sense of belonging significantly reduced PD [60]. Only the results of the US study are comparable to those of our unhoused participants, as it applied the same K6 scale. The lower SPD prevalence in the US likely reflects the differences in underlying socioeconomic conditions between the US and Vietnam.

In line with the findings of the present study, the prevalence of PD is reported to be higher in women than in men in most countries under normal circumstances [20, 61, 62] and during the COVID-19 pandemic [63–65]. Drapeau et al. offer three alternative hypotheses to explain the higher prevalence of PD among women: (1) PD may be partly attributable to gender-related personality traits or biological factors; (2) in most societies, women are either more exposed or more vulnerable to the risk factors associated with PD; and (3) the expression of emotions varies between genders. Nevertheless, despite being the subject of numerous studies, the gender difference in PD remains largely unexplained [20].

Labour exploitation was significantly associated with SPD among the participants. Abusive (authoritarian and aggressive) supervision has been reported to cause PD in affected workers [66]. Several studies highlight the major impact of violence or harassment at work, either by colleagues or supervisors, on workers’ PD [67–70]. Labour inspections and unionisation are potential measures to combat labour exploitation. However, labour inspection in Vietnam faces challenges due to an insufficient number of inspectors, resulting in many enterprises not being adequately inspected [71], raising questions about the feasibility and effectiveness of addressing labour exploitation through strengthening inspection. A more feasible approach may lie in the promotion of occupational safety and health (OSH). The Labour Code and Government Decree No. 6 require enterprises to implement all necessary measures for OSH [72]. The Ministry of Health (MOH) and local health bureaus conduct inspections related to occupational health [72], making this approach an entry point through the health sector. This has the potential to influence the chain of command within the Ministry of Home Affairs, which oversees labour issues since the absorption of the Ministry of Labour, Invalids, and Social Affairs (MOLISA) as part of the January 2025 restructuring.

Finally, indebtedness significantly increased the likelihood of SPD, and most of the borrowers were taking loans from moneylenders at interest. Illegal lending practices are common in Vietnam, and loan sharking is often disguised through seemingly lawful moneylenders, pawnshops, social media ads, or street posters. Charging exorbitant interest rates is a crime and is often linked to other illegal activities, such as gang involvement, money laundering, or violence [73]. Loan sharks targeting individuals with unstable incomes are prevalent [74]. Law enforcement may be hesitant or unable to investigate or prosecute these moneylenders, as loans are often made without formal documentation [73]. Despite these challenges, stricter regulation of loan sharks is necessary. In 2020, the Vietnam National Bank issued a circular facilitating debt rescheduling, interest and fee exemptions, or reductions to assist borrowers impacted by the COVID-19 pandemic [40]. Such policies may help reduce the financial and psychological burdens on populations with unstable housing and debt. However, these measures may be difficult to enforce with informal moneylenders. In addition to cracking down on predatory lending, promoting financial inclusion is essential. This is especially important as 26% of our participants were engaged in small businesses (e.g., selling lottery tickets) and needed access to business capital to survive, whereas approximately 79% of people in Vietnam do not have access to formal financial services [75].

The major findings of this study can be logically interpreted with reference to existing theoretical models, namely, the Social Determinants of Health (SDH) and the stress process models. The Commission on Social Determinants of Health (CSDH) presented a comprehensive framework for the SDH. This model consists of three key elements: (1) the socioeconomic and political context, (2) structural determinants and socioeconomic position, and (3) intermediary determinants, including material, behavioural, biological, and psychosocial factors [76]. Among the significant factors associated with SPD in this study, gender corresponds to the second and third elements. Labour exploitation and indebtedness primarily correspond to the third element as psychosocial factors and partly to the first element regarding labour and financial policies. The stress process model proposed by Turner [77] assumes that social conditions, including gender, lead to stress exposures, such as labour exploitation and indebtedness, which ultimately affect physical, mental, and overall health. Both social resources, such as social support and networks, and personal resources, such as a sense of control and self-esteem mediate this process.

The present study has several limitations. First, because we applied non-probability sampling methods without comprehensive sampling frames for both slum dwellers and unhoused individuals—due to the high proportion of unregistered residents at their living locations and their highly mobile lifestyle—we cannot estimate the prevalence of SPD in either group with precise statistical inference. Nor can we generalise the associations between SPD and explanatory variables to all residents living in slums serviced by SCDI or to all unhoused individuals sleeping in areas covered by SCDI. This limitation arises from potential bias introduced by non-probability sampling. The slum-dwelling sample is systematically more representative of those who agreed to participate and those who were present in their homes at the time of the survey. Regarding the former, since individuals with poor mental health may be less likely to agree to participate, the prevalence of SPD is likely underestimated. As for the latter, presence or absence at home during the survey is unlikely to be strongly related to SPD. Similarly, the unhoused sample is systematically more representative of those who sought care provided by SCDI. Since individuals with poor mental health may be less likely to seek care, the prevalence of SPD is also likely underestimated. Furthermore, our findings cannot be generalised to all slum dwellers and unhoused individuals in HCMC or other cities in Vietnam.

Second, we did not comprehensively explore the personal resources as risk and protective factors related to SPD. The risk and protective factors for PD can be categorised into three groups: (1) sociodemographic factors; (2) stress-related factors; and (3) personal resources [35]. We thoroughly examined categories 1 and 2 in terms of variables related to socioeconomic vulnerabilities, including housing status. However, for category 3, we only investigated income and education, omitting other external resources, such as social support and social networks, as well as internal resources such as self-esteem and a sense of control over one’s life.

Despite these limitations, this study provides a valuable snapshot of people with unstable housing in HCMC, who are highly marginalised and extremely difficult to reach. As they are not only highly mobile and isolated but also distrustful of outsiders, this study was only possible after years of trust-building through sustained service provision by the SCDI.

## Conclusions

The major findings of this study highlight the mental health needs and the associated socioeconomic and sociopolitical stressors among populations experiencing housing instability in rapidly growing mega-cities in low- and middle-income countries. They also make a substantial contribution to both service delivery and advocacy policies for SCDI and local authorities. The prevalence of SPD among populations with unstable housing, including slum dwellers and unhoused individuals in HCMC, was significantly higher than that of the general population in various countries. Given the high prevalence of psychological distress, mental health interventions targeted at these populations are urgently needed. SPD was significantly associated with female gender as well as labour and economic exploitation. In terms of labour exploitation, policy interventions, particularly from an occupational health perspective, are necessary. To address economic exploitation through debt, it is essential to implement concurrent efforts to crack down on predatory lending and promote financial inclusion, ensuring access to the financial services necessary for maintaining small businesses. Future research should employ a more rigorous probability-based and random sampling methodology and examine social resources, such as social support and social networks, as well as internal resources, such as self-esteem and a sense of control over one’s life, as potential factors that may either exacerbate or mitigate SPD.

## Data Availability

The dataset generated and/or analysed during the current study is not publicly available due to the inclusion of the names of the interviewers but is available from the corresponding author upon reasonable request.

## Abbreviations

BSI-18: Brief Symptom Inventory-18
CES-D: Center for Epidemiological Studies Depression Scale
COVID-19: Coronavirus disease 2019
DSM: Diagnostic and Statistical Manual of Mental Disorders
DSM-IV: Diagnostic and Statistical Manual of Mental Disorders, fourth edition
GHQ-12: General Health Questionnaire-12
HCMC: Ho Chi Minh City
HIV: Human immunodeficiency virus
ICD: International Classification of Diseases
ID: Identification
IRB: Independent Review Board
ISDS: Institute for Social Development Studies, Vietnam
K10: Kessler Psychological Distress Scale (10-item version)
K6: Kessler Psychological Distress Scale (6-item version)
METI: Ministry of Economy, Trade and Industry, Japan
MEXT: Ministry of Education, Culture, Sports, Science and Technology, Japan
MOH: Ministry of Health, Vietnam
MOHLW: Ministry of Health, Labour and Welfare, Japan
MOLISA: Ministry of Labour, Invalides and Social Affairs, Vietnam
NCGM: National Center for Global Health and Medicine, Japan
OR: Odds ratio
OSH: Occupational safety and health
PD: Psychological distress
SCDI: Center for Supporting Community Development Initiatives
SD: Standard deviation
SPD: Serious psychological distress
SPSS: Statistical Package for the Social Sciences
US: The United States (of America)
VND: Vietnam dong
